# Optimization of the growth conditions through response surface methodology and metabolomics for maximizing the auxin production by *Pantoea agglomerans* C1

**DOI:** 10.3389/fmicb.2023.1022248

**Published:** 2023-03-08

**Authors:** Francesca Melini, Francesca Luziatelli, Paolo Bonini, Anna Grazia Ficca, Valentina Melini, Maurizio Ruzzi

**Affiliations:** ^1^Department for Innovation in Biological, Agrofood and Forest Systems, University of Tuscia, Viterbo, Italy; ^2^CREA Research Centre for Food and Nutrition, Rome, Italy; ^3^OloBion–OMICS LIFE LAB, Barcelona, Spain

**Keywords:** *Pantoea agglomerans*, metabolomics, response surface methodology, postbiotics, plant growth-promoting rhizobacteria, auxin

## Abstract

**Introduction:**

The fermentative production of auxin/indole 3-acetate (IAA) using selected *Pantoea agglomerans* strains can be a promising approach to developing novel plant biostimulants for agriculture use.

**Methods:**

By integrating metabolomics and fermentation technologies, this study aimed to define the optimal culture conditions to obtain auxin/IAA-enriched plant postbiotics using *P. agglomerans* strain C1. Metabolomics analysis allowed us to demonstrate that the production of a selected.

**Results and discussion:**

Array of compounds with plant growth-promoting- (IAA and hypoxanthine) and biocontrol activity (NS-5, cyclohexanone, homo-L-arginine, methyl hexadecenoic acid, and indole-3-carbinol) can be stimulated by cultivating this strain on minimal saline medium amended with sucrose as a carbon source. We applied a three-level-two-factor central composite design (CCD) based response surface methodology (RSM) to explore the impact of the independent variables (rotation speed and medium liquid-to-flask volume ratio) on the production of IAA and IAA precursors. The ANOVA component of the CCD indicated that all the process-independent variables investigated significantly impacted the auxin/IAA production by *P. agglomerans s*train C1. The optimum values of variables were a rotation speed of 180 rpm and a medium liquid-to-flask volume ratio of 1:10. Using the CCD-RSM method, we obtained a maximum indole auxin production of 208.3 ± 0.4  mg IAA_equ_/L, which was a 40% increase compared to the growth conditions used in previous studies. Targeted metabolomics allowed us to demonstrate that the IAA product selectivity and the accumulation of the IAA precursor indole-3-pyruvic acid were significantly affected by the increase in the rotation speed and the aeration efficiency.

## Introduction

World population growth (UN, 2017), the effect of climate change on the global food systems (Myers et al., 2017), the COVID-19 pandemic (Mardones et al., 2020), and the impact of the war in Ukraine on the global energy market, the grains supply, and the food prices (Toffelson, 2022) are critical challenges for today’s agriculture. Within this framework, the use of plant biostimulants has generated significant interest, and, among biostimulants, plant growth-promoting (rhizo)bacteria (PGPR or PGPB) look very promising to develop more efficient and sustainable production methods (Calvo et al., 2014; Ruzzi and Aroca, 2015; Rouphael and Colla, 2018).

PGPB are a heterogeneous group of endophytic and epiphytic bacteria associated with plant tissues and plant surfaces which can positively affect the health and growth of plants (Glick, 2012; de Souza et al., 2015). They can increase crop tolerance against abiotic stresses and improve nutrient use efficiency, plant health, productivity, and yield at different stages (Bulgari et al., 2015; Toscano et al., 2018). They exert beneficial effects on plants through direct and indirect mechanisms, which range from changes in hormonal content to the production of volatile organic compounds, the increase in nutrient availability, or the enhancement of abiotic stress tolerance (Ruzzi and Aroca, 2015; Oleńska et al., 2020).

So far, the European Union Regulation (EU) (2019)/1009 authorizes, as “microbial biostimulants,” the use of bacteria belonging to only three taxonomic groups, namely, *Azotobacter*, *Azospirillum*, and *Rhizobium* [European Union Regulation (EU), 2019/1009]. These constraints preclude and limit the development of novel biostimulants that contain PGPR belonging to different taxonomic groups. A microorganism belonging to Risk Group I, according to the European Parliament Directive 2000/54/EC, is unlikely to cause disease in humans, animals, or plants, which does not mean that its presence could not affect the microbial biodiversity or, under critical life circumstances (e.g., an impaired health status), the health of a higher organism.

In many cases, in both humans and plants, health-promoting traits are associated with the production of soluble and volatile metabolites secreted in the culture medium. Recently, in the human sector, it has been introduced a new class of dietary biotics, the postbiotics or metabiotics. In 2021, the panel of experts designated by the International Association of Probiotics and Prebiotics (ISAPP) defined these products as a “preparation of inanimate microorganisms and/or their components that confer a health benefit on the host” containing “inactivated microbial cells or cell components, with or without metabolites, that contribute to observed health benefits” (Salminen et al., 2021). Bioactive compounds generated by fermentation using PGPR can represent a valuable approach to developing a novel class of biostimulants with higher safety and a lower environmental impact and studying the host plant response to PGPR’s metabolites at the molecular level.

Regarding the production and activation of phytohormones by which plant growth is prompted, indole-3-acetic acid (IAA) is the primary auxin endogenously synthesized by PGPR with an essential effect on plant growth and development processes, such as cell elongation and organogenesis, and tropic responses (Woodward and Bartel, 2005; Teale et al., 2006; Halliday et al., 2009). Auxin contributes to root initiation, helps loosen plant cell walls to release exudates, stimulates root hair overproduction, increases lateral root formation, acts as a signaling molecule to both the plant and bacteria, aids in root elongation (Kasahara, 2016), and in the developmental response to stress (Bielach et al., 2017) and other environmental stimuli (Zwiewka et al., 2019). In plant tissues, the auxin responses are concentration-dependent, and different cells respond very differently to exogenous auxins (Paponov et al., 2008).

Indole auxin production by microbial isolates changes among different species and strains of the same species and is influenced by culture conditions, growth stage, and substrate availability (Duca et al., 2014). The presence of tryptophan, vitamins, salt, pH, temperature, carbon source, nitrogen source, and growth phase contribute to regulating auxin/IAA biosynthesis (Ryu and Patten, 2008; Swain and Ray, 2008; Apine and Jadhav, 2011; Bharucha et al., 2013; Peng et al., 2014; Luziatelli et al., 2020a). Plant exudates or specific compounds present in the rhizosphere are among the factors influencing bacterial auxin/IAA biosynthesis. Therefore, the mechanisms involved in the control of biosynthesis of IAA and indole-related compounds require a complete understanding.

Various IAA biosynthetic pathways have been proposed in plant-associated bacteria (Spaepen and Vanderleyden, 2011; McClerklin et al., 2018). Tryptophan is a major effector of IAA biosynthesis, considering most IAA biosynthesis pathways begin with tryptophan. Tryptophan-dependent pathways comprise the indole-3-acetamide (IAM), indole-3-pyruvic acid (IPyA), indole-3-acetonitrile (IAN), tryptamine (TAM), and tryptophan side-chain oxidase (TSO) pathways (Duca et al., 2014; Di et al., 2016). Organic nitrogen sources have been observed to promote auxin/IAA production more than inorganic nitrogen sources, and this may be due to the increased availability of tryptophan from proteins.

The application of the genus *Pantoea* as a biocontrol agent has been increasingly investigated, and exciting results have been obtained. Strains belonging to *Pantoea agglomerans* species are frequently found in association with plant hosts (Walterson and Stavrinides, 2015). These microorganisms are agronomically relevant for their plant-growth-promoting (PGP) features, biocontrol activity, and involvement in plant disease management (Dutkiewicz et al., 2016).

The PGP activities of *P. agglomerans* strains have been investigated through different application modes and under diverse experimental conditions (e.g., glasshouse, field, pots, greenhouse, etc.; Nunes et al., 2002; Feng et al., 2006; Venkadesaperumal et al., 2014; Moustaine et al., 2016; Paredes-Páliz et al., 2016; Kamran et al., 2017; Ozaktan et al., 2017; Xie et al., 2017; Saia et al., 2020; Luziatelli et al., 2020a,b).

Recently, we demonstrated by comparative genomic analysis high levels of conservation within genes of the IPyA pathway in this species. Independently from the natural environment from which the strain was isolated, in most of the sequenced genomes of isolates belonging to *P. agglomerans* species (45 out of 50), the genes encoding enzymes of the IPyA pathway share an identity higher than 95% over the full length of the sequence (Luziatelli et al., 2020a). This high sequence conservation has never been reported for IAA genes belonging to other species underlining that the production of auxin/IAA plays an important role in the interaction between *P. agglomerans* and host plants (Luziatelli et al., 2020a).

Optimizing the physical and chemical environmental factors that affect a fermentation process (such as temperature, pH, aeration, and medium composition) is crucial for maximizing the yield of a specific bioproduct (Schmidt, 2005; Singh et al., 2017). Several optimization approaches can be used for this purpose, but the most common is the one-factor-at-a-time (OFAT). This approach offers the advantage of measuring whether a factor has any effect on the microbial production of the desired product and affects the metabolic traits of the producing strain (Singh et al., 2011). For the same reason, classical studies on comparative metabolomics mainly focus on the metabolic changes associated with the variation of an environmental factor only (Putri et al., 2013). The major downside of using OFAT is that it requires many experimental runs and is time-consuming and expensive, particularly in fermentation processes (Panda et al., 2007) and metabolomics analysis. Moreover, OFAT is not adequate for investigating the interaction between different variables. This limitation can be overcome using a statistical approach such as Response Surface Methodology (RSM). The latter is a useful multivariate statistical tool that allows one to design experiments, construct models, evaluate the combined effect of two or more parameters, and search for optimum conditions for desirable responses (Bezerra et al., 2008; Breig and Luti, 2021). RSM has been widely exploited to optimize fermentation processes, but surprisingly it has not been used in combination with metabolomics. This is probably because metabolomics (particularly untargeted metabolomics) is used when no *a priori* metabolic hypothesis is available (Gertsman and Barshop, 2018). At the same time, a proper selection of the factors ranges for designing the experimental matrix needs prior knowledge of the genetics and physiology of the microorganism (Breig and Luti, 2021).

Few studies are available on the process optimization of indole auxin production in *P. agglomerans* and other PGPB (Apine and Jadhav, 2011; Chaiharn and Lumyong, 2011; Balaji et al., 2012; Bharucha et al., 2013; Tallapragada et al., 2015; Bhutani et al., 2018; Myo et al., 2019; Luziatelli et al., 2021). Furthermore, there are no reports on the effect of the medium aeration on the *in vitro* production of indole auxin and, except for Luziatelli et al. (2020a), none of the above-mentioned studies has evaluated the impact of the culture conditions stimulating IAA synthesis on the production of other metabolites involved in pathways associated with PGP traits. *In vitro* studies carried out on *Pyrus communis* shoots demonstrated that *P. agglomerans* strain C1 produces metabolites that act in synergy with auxin, accelerating the adventitious roots (AR) formation when auxins are present, increasing the number and length of AR primordia that develop from stems, and modifying the response of the pear plant to exogenous auxins (Luziatelli et al., 2020c). These data suggest that in the development of an auxin-enriched plant postbiotic, the auxin/IAA concentration is not the unique parameter that should be considered.

The main goal of the present study was to optimize the auxin production in *P. agglomerans* strain C1 and evaluate the effect of the optimum conditions on the metabolites secreted in combination with auxin. To this aim, we investigated, by central composite design (CCD) combined with RSM, the effect of aeration efficiency and medium composition on auxin production. In combination with RSM-CCD, we also used a targeted and untargeted metabolomics approach to evaluate the effect of the growth conditions on the auxins profile and the production of metabolites potentially involved in the plant-microbe interaction.

## Materials and methods

### Bacterial strain and culture media

*Pantoea agglomerans* strain C1 was previously isolated from the phyllosphere of lettuce (*Lactuca sativa* L.) plants treated with vegetal-derived protein hydrolysates (Luziatelli et al., 2016, 2019a). Working cultures were routinely grown at 25°C on Lennox broth (LB; composition per liter: tryptone 10 g, yeast extract 5 g, NaCl 5 g; Lennox, 1955); and stored at −80°C as stock cultures, using glycerol (20% w/w) as a cryoprotectant. For auxin production, the strain was cultivated in a medium amended with 4 mM tryptophan (Trp), using either LB or a saline M9-sucrose broth (YES; composition per liter: M9 minimal salts 1× yeast extract 5 g, and sucrose 5 g). Tryptophan was prepared as 40 mM stock solution in mildly alkaline water, filter sterilized (0.22 μm), and stored in the dark at 4°C. All culture mediums were sterilized by autoclaving at 121°C for 20 min. Tryptone and yeast extract were purchased from BD Difco (Thermo Fisher Scientific, MA, United States), and sucrose and chemicals were procured from Sigma-Aldrich (Merck KGaA, Darmstadt, Germany).

### Culture conditions

Pre-cultures were prepared by inoculating 50 mL of LB with 500 μL of a frozen (−80°C) stock culture. Cells were grown at 25°C in agitation (150 rpm) until the late exponential phase of growth (10^8^ CFU/mL) using an INFORS HT Multitron incubator (Infors AG, Bottmingen, Switzerland). Aliquots of the pre-culture were transferred into 250 mL Erlenmeyer flasks containing YES medium broth amended with Trp to obtain an initial OD_600_ of 0.1 and were incubated at 25°C for 24 h. The total medium volume (mL) per flask and the agitation speed (rpm) were varied according to the experimental design described below (Table 1). To avoid any interference due to the flask geometry and type of closure, all experiments were carried out using (i) 500 mL Erlenmeyer flasks for strain pre-activation experiments, (ii) certified 250 mL narrow-neck Erlenmeyer flasks corresponding to the ISO 1773:1997 specifications (external diameter of bottom and neck of 85 ± 2 and 34 ± 1.5 mm, respectively), and (iii) T-32 silicon plugs with bubble sponge, for optimization of auxin/IAA production. Culture volume and vessels affect physiological responses and genetic stability in several microorganisms, such as *Escherichia coli* (Kram and Finkel, 2014; Gross et al., 2020).

**Table 1:** Range and levels of experimental variables of the two sets of experiments.

Factor	Experimental design no. 1			Experimental design no. 2		
	*Actual levels of coded factors*			*Actual levels of coded factors*		
	−1	0	+1	−1	0	+1
Rotation speed (rpm)	100	140	180	160	180	200
Medium volume (mL)	25	62.5	100	10	25	40

### Factorial design for cell growth

**Figure 1:**
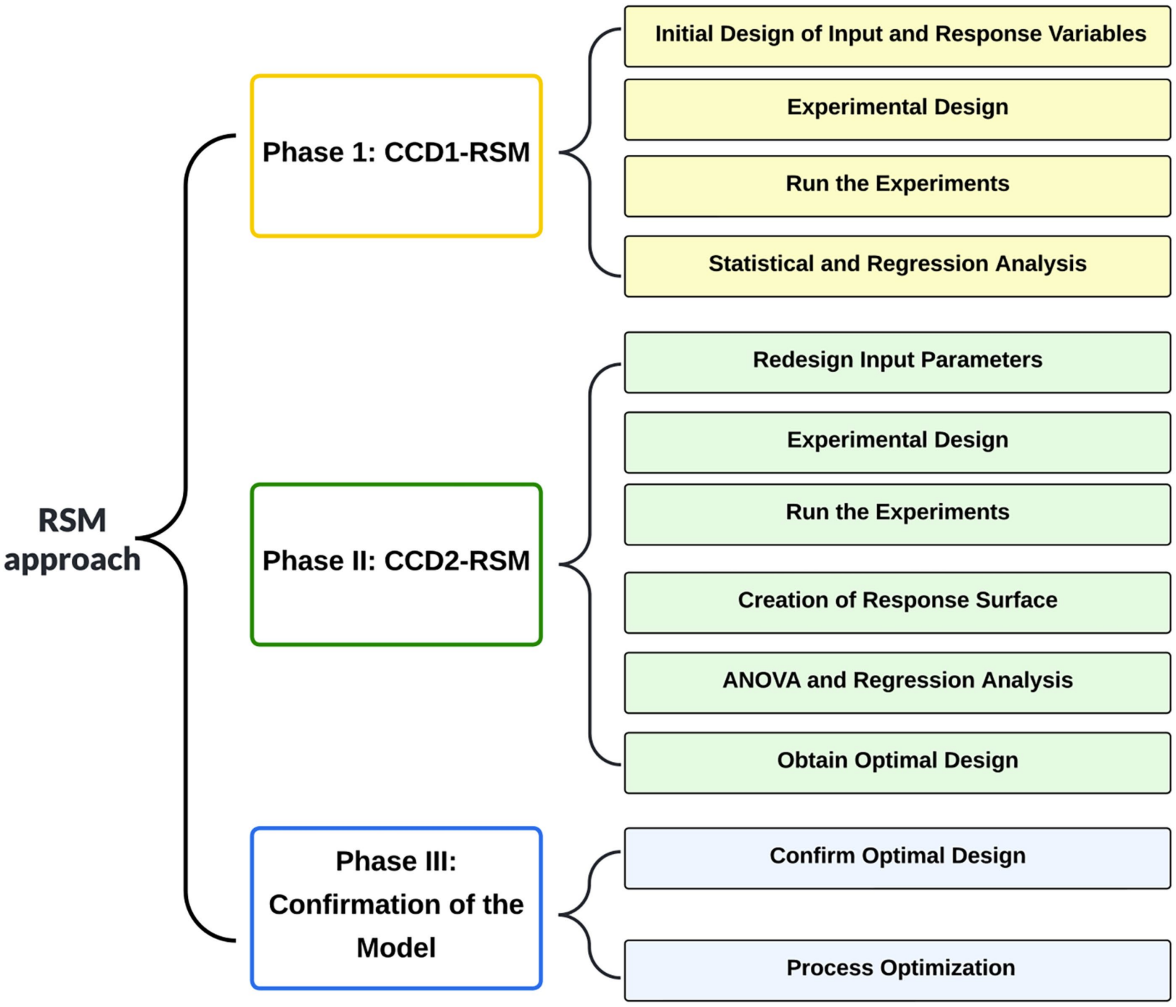
Flowchart of the Response Surface Methodology (RSM) modeling approach.

The experimental design was established by using the DOE software package MODDE ver. 12 (Sartorius, Göttingen, Germany) as reported elsewhere (Luziatelli et al., 2019b). The Response Surface Methodology (RSM) method was used to determine the optimum level of each variable, and the combination thereof allowed the maximum productivity of auxin. Rotation speed (rpm) and liquid medium volume (mL) were set as factors. Cell biomass (OD_600_) and auxin production (mg/L) were set as responses. Figure 1 shows the procedure used to develop the RSM design matrix and find optimum values for the input process parameters.

In the first experimental design, two ranges of values were set for the relevant factors (i.e., rotation speed and medium volume), as shown in Table 1. Each independent variable was tested at three levels, indicated as −1 (low level), 0 (central level), and + 1 (high level).

According to the first Central Composite Design (CCD1), 20 experimental runs were executed, with four center value replications, and the observations were fitted to the following second-order polynomial model:

*Y* = β_0_ + β_1_(*X*_1_) + β_2_(*X*_2_) + β_11_(*X*_1_^2^) + β_22_(*X*_2_^2^) + β_12_(*X*_1_*X*_2_).

Where: *Y* is the dependent variable (indole auxins production); *X*_1_ and *X*_2_ are the coded independent variables (rotation speed and medium volume); β_0_ is the regression coefficient at the center point; β_1_ and β_2_ are the linear coefficients; β_11_ and β_22_ are the quadratic coefficients; β_12_ is the second-order interaction coefficient. The developed regression model was evaluated by analysis of variance (ANOVA) and *p*- and *F*-values. The quality of the fit of the polynomial model equation was expressed by the coefficient of determination, *R^2^*. The significance of all terms in the polynomial was judged statistically by computing the *F* value at a probability (*p*) of 0.05. The regression coefficients were used to make statistical calculations to generate response surface curves from the regression models. To test the model accuracy, *R^2^* and adjusted *R^2^* were estimated.

Based on the results of the first round of experiments, a second experimental design was set by MODDE ver. 12 by centering on the agitation speed of 180 rpm and the volume of 25 mL. The RSM-CDD (CDD2) comprised a total of 18 experimental runs. The two ranges of value for the relevant factors set in the second experimental design are shown in Table 1.

All experiments were carried out in duplicate in the standard conditions previously defined: growth in YES-Trp medium for 24 h; cultivation in a rotary shaker at 25°C using 250 mL narrow-neck Erlenmeyer flasks with T-32 silicon plugs.

### Spectrophotometric estimation of indole auxins

Auxin production was measured in the cell-free spent medium using a colorimetric assay with Salkowski’s reagent (Patten and Glick, 2002). Briefly, cell culture was centrifuged at 10000 rpm for 10 min in a multispeed centrifuge (Thermo Fisher Scientific, Waltham, MA, USA), and the supernatant was recovered and filter-sterilized (0.22 μm). An aliquot (1 mL) of the supernatant, diluted as needed, was mixed vigorously with 2 mL of Salkowski’s reagent (0.5 M FeCl_3_, 35% v/v HClO_4_), and the mixture was incubated at room temperature for 20 min in the dark. The developed pink color was read at 530 nm. Total indole auxins were quantified by using a calibration curve of pure indole-3-acetic-acid (Sigma–Aldrich, Milan, Italy) as a standard. Data were expressed as μg IAA equivalents (IAA_equ_) per mL of liquid culture. No reaction product was determined using not inoculated medium amended with tryptophan as a control.

### Footprinting of extracellular metabolites

The effect of the growth medium on the extracellular metabolites was tested by cultivating *P. agglomerans* strain C1, at optimal growth temperature (30°C), on LB and YES medium supplemented with Trp (4 mM). Exhausted liquid broth samples (1 mL) were extracted in 5 mL of cold (−20°C) acidified (0.1% HCOOH) 80:20 methanol:water using an Ultra-Turrax (Ika T-25, Staufen, Germany), centrifuged at 1,200 rpm and filtered through a 0.2 μm cellulose membrane. The analysis was carried out on an Agilent 6550 Q-TOF with ESI source (Agilent Technologies, Santa Clara, CA, United States), coupled with an Agilent 1290 UHPLC, as reported elsewhere (Luziatelli et al., 2020a). 2 μL of each sample were injected on a reversed-phase Acquity ethylene-bridged hybrid (BEH) HILIC C18 column (100 mm × 2.1 mm, 1.7 μm, Waters, Milford, MA, USA). A pooled quality control was obtained by mixing 10 μL of each sample and acquired in tandem mass spectroscopy mode using iterative function five consecutive times to increase the number of compounds with associate MS2 spectra.

The raw data were processed with the procedure described by Blaženović et al. (2019). Compounds identification was based on accurate mass and isotopic patterns and expressed as an overall identification score. Chemical Similarity Enrichment Analysis (ChemRICH) was performed, as described by Barupal and Fiehn (2017), and statistical significance *p*-values were obtained by self-contained Kolmogorov–Smirnov tests.

### Auxin profiling

In targeted metabolomics, chromatographic separation, MS, and MS/MS acquisition parameters were carried out following the FiehnLab HILIC protocol, as previously described (Blaženović et al., 2018; Teleki and Takors, 2019). The injection volume was 2 μL, whereas Q-TOF was operated in positive SCAN mode (100–1,700 m/z + range) and extended dynamic range mode. All samples were injected in three biological replicates with three technical replicates each (total 9 injections/sample). Deconvolution, mass and retention time alignment, normalization, blank filtering, and identification were carried out using the Agilent Mass Hunter Quantitative Analysis software for Q-TOF version B.09. Standards for quantification and identification of Trp, IAA, and IPyA were purchased from Sigma-Aldrich. Three dilutions were prepared in duplicate with a concentration of 10, 100, 500, and 1,000 ppm from each standard to calculate the calibration curve, reaching an *R^2^* of 0.999 in all compounds.

### Statistical analysis

Differences between treatment groups were compared using the One-way analysis of variance (ANOVA) test, followed by Tukey’s honestly significant difference (HSD) test with significance set at *p* < 0.05.

## Results

### Effect of growth medium composition on exometabolome

**Figure 2:**
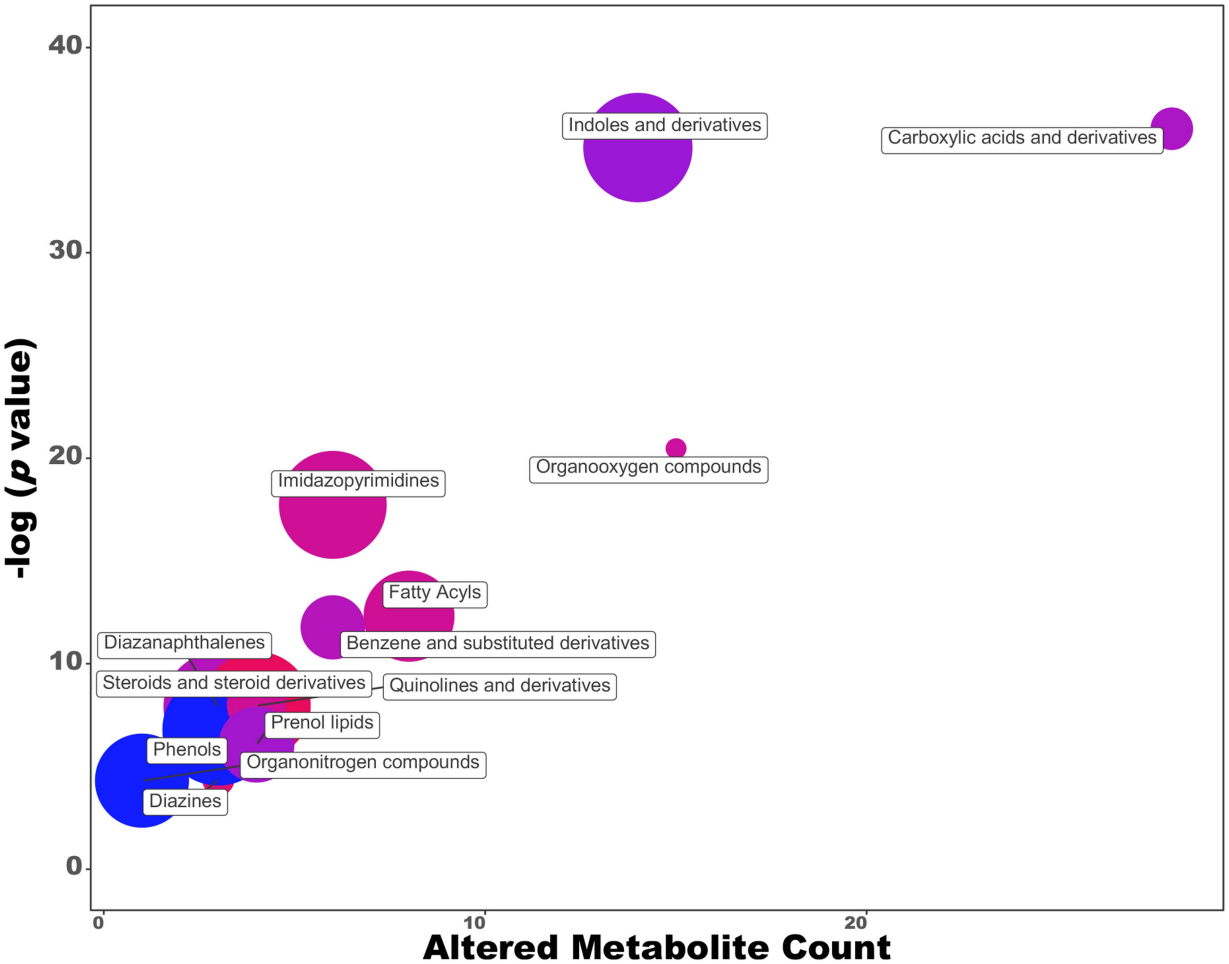
ChemRich analysis of exometabolome of *Pantoea agglomerans* C1 grown on YES vs. LB medium. Red clusters are associated with higher outcomes, and the blue ones are associated with lower outcomes.

As demonstrated in previous work (Luziatelli et al., 2020a), the production of IAA by *P. agglomerans* strain C1 is affected by the carbon source and the incubation temperature. Higher auxin levels were obtained by growing C1 strain on an M9 saline medium with sucrose (YES) as a carbon source under suboptimal temperature conditions (25°C; Luziatelli et al., 2020a). In this work, we comprehensively characterized strain C1 exometabolome of cultures grown on a medium amended with tryptophan as an auxin/IAA precursor. UHPLC-ESI-Q-TOF-MS analysis of the exhausted growth medium revealed a total of 204 features: 16 were more than two-fold higher, and 54 were more than two-fold lower, changing the cultivation medium from LB + Trp to YES+Trp (Figure 2; Supplementary Table S1). Comparing the metabolomic datasets of cultures grown on LB and YES medium by Chemical Similarity Enrichment Analysis (ChemRICH), we observed a significant increase in IAA (5.4 folds), indole-3-carbinol (3.9 folds), quinoline (2.8 folds), cyclohexane derivatives shifting from LB to YES (Supplementary Table S1). Under the same conditions, a significant decrease of IAA precursors (i.e., indole-3-acrylic acid, indole acetaldehyde, indole-3-acetamide, and indole-3-carboxylic acid) was observed (Supplementary Table S2). Growth in the YES medium also determined a significant variation in the hypoxanthine/xanthine ratio due to a 4.8-fold increase in hypoxanthine concentration and a 105-fold decrease in xanthine. Other metabolites, whose production was stimulated on YES medium, included compounds with antimicrobial activity such as the β-lactam antibiotics NS-5 and 2-hydroxyethylclavam, the cyclohexanone, the homo-L-arginine, and the methyl hexadecanoic acid.

### Effect of oxygen availability on biomass and auxin yield

**Figure 3:**
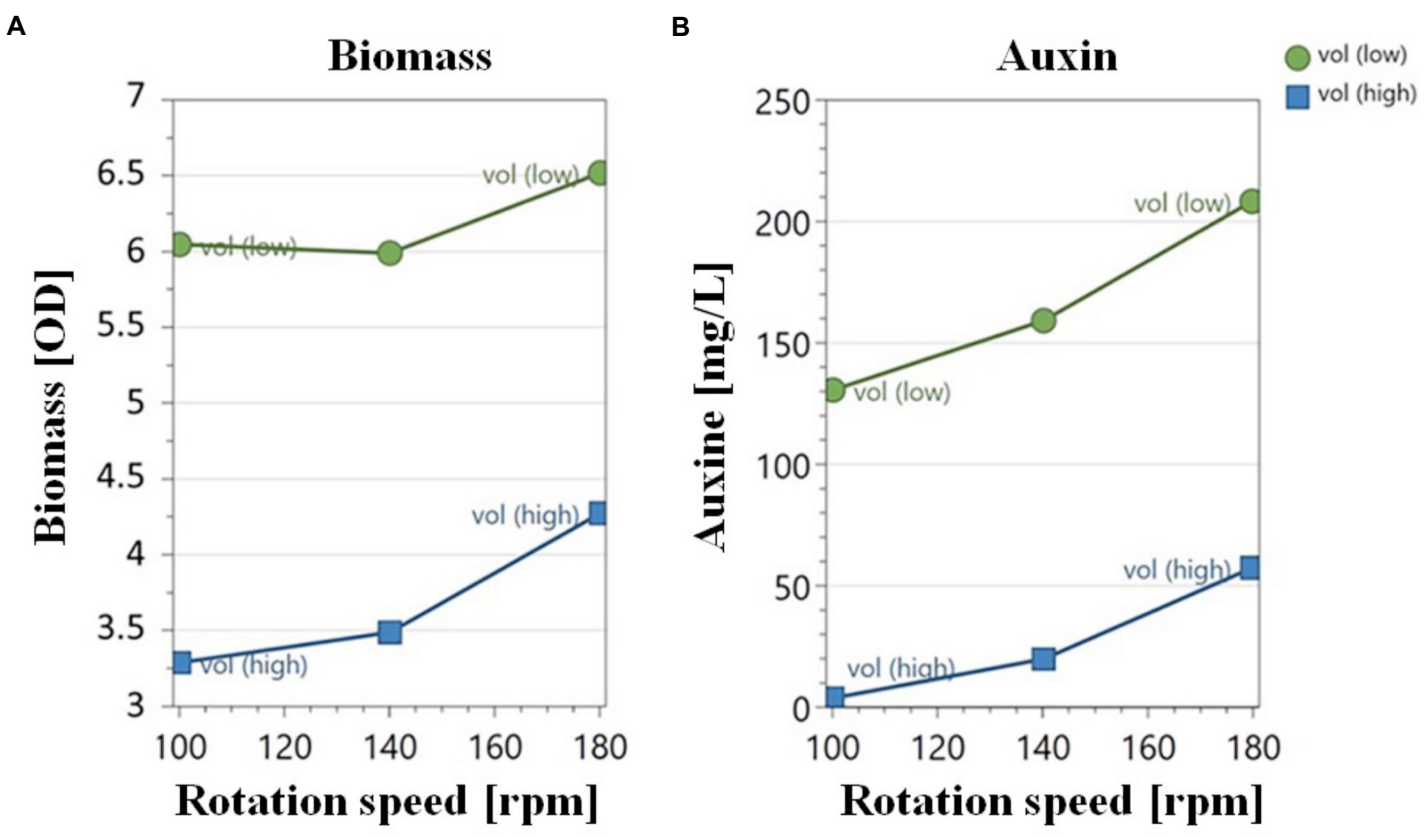
Effect of the rotation speed on biomass **(A)** and auxin yield **(B)** by *Pantoea agglomerans* strain C1 in shake-flask cultivation experiments carried out at a liquid-to flask V*_m_*/V*_f_* ratio of 1:2.5 (low) and 1:10 (high).

The effect of oxygen availability on cell biomass and auxin production was evaluated on YES medium using a shake-flask methodology by varying medium liquid-to-flask volume ratio (V*_m_*/V*_f_*) and rotation speed (rpm). Data reported in Figure 3A indicated that an increase in the biomass yield (OD_600_) occurred upon increasing the gas–liquid interfacial area by either changing the V*_m_*/V*_f_* ratio (from 1:2.5 to 1:10) or the rotation speed (from 100 to 180 rpm). OD_600_ varied between 3.4 ± 0.0 and 6.4 ± 0.1, and the highest biomass production (OD_600_ = 6.45) was achieved at 180 rpm and V*_m_*/V*_f_* ratio of 1:10 (Table 2, run 4). At all V*_m_*/V*_f_* ratio values, no significant change in the OD_600_ was measured by increasing the rotation speed from 100 to 140 rpm. As expected, at 180 rpm, the increase in the biomass yield was more marked (OD_600_ from 4.2 ± 0.0 and 6.4 ± 0.1) by increasing the V*_m_*/V*_f_* ratio from 1:2.5 to 1:10 (Figure 3A).

**Table 2:** Experimental design No. 1 and results of CCD1-RSM.

Run	Factor		Volume ratio	Observed response		Predicted response		Auxin spec. productivity (mg IAA_equ_ /L/OD_600_)
	*Rotation speed (rpm)*	*Medium volume (mL)*	V*_m_*/V*_f_*	Biomass (OD_600_)	Auxin (mg IAA_equ_/L)	Biomass (OD_600_)	Auxin (mg IAA_equ_/L)	
1	(0) 140	(−1) 25.0	1:10	6.11	163.21	5.99	158.97	26.71
2	(−1) 100	(−1) 25.0	1:10	6.12	133.31	6.05	130.30	21.78
3	(+1) 180	(+1) 100.0	1:2.5	4.24	61.05	4.27	56.95	14.40
4	(+1) 180	(−1) 25.0	1:10	6.45	209.82	6.52	208.21	32.53
5	(−1) 100	(−1) 25.0	1:10	6.04	125.33	6.05	130.30	20.75
6	(+1) 180	(0) 62.5	1:4	5.37	98.73	5.10	103.92	18.39
7	(0) 140	(0) 62.5	1:4	4.18	47.73	4.45	60.93	11.42
8	(−1) 100	(+1) 100.0	1:2.5	3.37	3.43	3.29	4.04	1.02
9	(0) 140	(+1) 100.0	1:2.5	3.42	3.13	3.29	4.04	0.92
10	(+1) 180	(+1) 100.0	1:2.5	4.18	52.77	4.27	56.95	12.62
11	(−1) 100	(−1) 25.0	1:10	6.10	157.22	5.99	158.97	25.77
12	(0) 140	(0) 62.5	1:4	4.76	76.92	4.45	60.93	16.16
13	(−1) 100	(0) 62.5	1:4	4.34	48.98	4.38	38.51	11.29
14	(+1) 180	(0) 62.5	1:4	5.25	109.76	5.10	103.92	20.91
15	(−1) 140	(0) 62.5	1:4	4.54	53.71	4.45	60.93	11.83
16	(−1) 140	(0) 62.5	1:4	4.17	61.28	4.45	60.93	14.70
17	(0) 140	(+1) 100.0	1:2.5	3.50	21.44	3.49	20.21	6.13
18	(−1) 100	(0) 62.5	1:4	4.15	31.50	4.38	38.51	7.59
19	(0) 140	(+1) 100.0	1:2.5	3.39	20.57	3.49	20.21	6.07
20	(+1) 180	(−1) 25.0	1:10	6.31	206.08	6.52	208.21	32.66

Coded (in parentheses) and real values are shown.

Data reported in Figure 3B indicated that the aeration efficiency of the culture medium, dependent upon the rotation speed and liquid-to-flask volume ratio, also affected the extracellular concentration and specific productivity of auxin. When the oxygen availability was lower (lower rotation speed and higher medium liquid-to-flask volume ratio), a lower amount of auxin was obtained. At all agitation regimes, higher auxin values were achieved using a 1:10 medium liquid-to-flask volume ratio (Figure 3B). The highest auxin titer (208.0 ± 2.0 mg of IAA_equ_/L) and specific productivity (32.6 ± 0.1 mg of IAA_equ_/L/OD_600_) mean values were obtained at 180 rpm with 25 mL of liquid culture in a 250 mL flask (run 4 and 20; Table 2).

### Optimization of auxins production by RSM

To evaluate more precisely the effect of oxygen availability on auxin yield, a second experimental design centered on the agitation speed of 180 rpm and the liquid-to-flask volume ratio of 1:10 was performed. Predicted and observed data of CCD No. 2 for biomass (OD_600_) and auxins production (mg/L) are reported in Table 3.

**Table 3:** Experimental design No. 2 and results of CCD2-RSM.

Run	Factor		Liquid-to-flask volume ratio	Observed Response	Predicted Response
	*Rotation speed (rpm)*	*Medium volume (mL)*	V*_m_*/V*_f_*	Auxin (mg IAA_equ_/L)	Auxin (mg IAA_equ_/L)
1	(−1) 160	(−1) 10	1:25	181.91	180.43
2	(+1) 200	(−1) 10	1:25	156.81	159.67
3	(−1) 160	(+1) 40	1:6.25	161.41	160.75
4	(+1) 200	(+1) 40	1:6.25	155.21	154.13
5	(−1) 160	(0) 25	1:10	192.42	197.35
6	(+1) 200	(0) 25	1:10	186.33	183.66
7	(0) 180	(−1) 10	1:25	190.51	186.49
8	(0) 180	(+1) 40	1:6.25	168.78	173.88
9	(0) 180	(0) 25	1:10	208.11	206.94
10	(−1) 160	(−1) 10	1:25	179.63	180.43
11	(+1) 200	(−1) 10	1:25	157.45	159.67
12	(−1) 160	(+1) 40	1:6.25	166.20	160.75
13	(+1) 200	(+1) 40	1:6.25	153.38	154.13
14	(−1) 160	(0) 25	1:10	195.51	197.35
15	(+1) 200	(0) 25	1:10	185.74	183.66
16	(0) 180	(−1) 10	1:25	186.89	186.49
17	(0) 180	(+1) 40	1:6.25	172.54	173.88
18	(0) 180	(0) 25	1:10	207.79	206.94

Coded (in parentheses) and real values are shown.

The results obtained with the experiments scheduled in the CCD2 confirmed that the highest auxin values (208.0 ± 0.2 mg of IAA_equ_/L) were achieved when the C1 strain was grown at 180 rpm with 25 mL of liquid culture (1:10 medium liquid-to-flask volume ratio) in a 250 mL flask (run 9 and 18; Table 3).

The experimental results were fitted to a second-order polynomial quadratic equation. A multiple correlation analysis was done, and the following equation was thus obtained:

$$Y=206.94-6.85\;X_{1}-6.31\;X_{2}-16.44\;X_{1}{}^{2}-26.76\;X_{2}{}^{2}+3.53\;X_{1}X_{2}$$

where Y was the predicted auxin concentration, and *X*_1_ and *X*_2_ were the coded values for medium volume and rotation speed, respectively.

Statistical analysis results, including the regression coefficient and *p*-values for linear, quadratic, and interaction effects, are shown in Table 4.

**Table 4:** Regression analysis of the full second-order polynomial model for optimization of auxins.

Model term	Coefficient estimated	Std. Errors	*p*-value	Confidence intervals (±)
Intercept	206.941	1.756	9.316 E-20	3.83
X_1_	−6.847	0.962	1.213 E-05	2.10
X_2_	−6.307	0.962	2.695 E-05	2.10
X_1_^2^	−16.437	1.665	4.129 E-09	3.63
X_2_^2^	−26.757	1.665	1.769 E-09	3.63
X_1_ X_2_	3.532	1.178	0.011	2.57

*R^2^* = 0.974, adj-*R^2^* = 0.964.

The multiple regression analysis of auxin production showed that the model was significant (*p* < 0.05) and did not present a lack of fit (*p* = 0.04). The regression-based determination of *R^2^* was evaluated to test the model equation’s fit. The *R^2^* value, which always ranges between 0 and 1, provided a measure of how the experimental factors and their interactions can explain much variability in the observed response values. In this study, the multiple coefficients of determination (*R^2^*) for auxin production were 0.974, which means that the model can account for 97.4% of the variability in the response. The adjusted *R^2^* of 0.964 corrects the *R^2^* value for the sample size and the number of terms in the model. The *Q^2^* value, which provides a qualitative measure of consistency between the predicted and original data, was equal to 0.943, indicating that the model has good predictive relevance.

**Figure 4:**
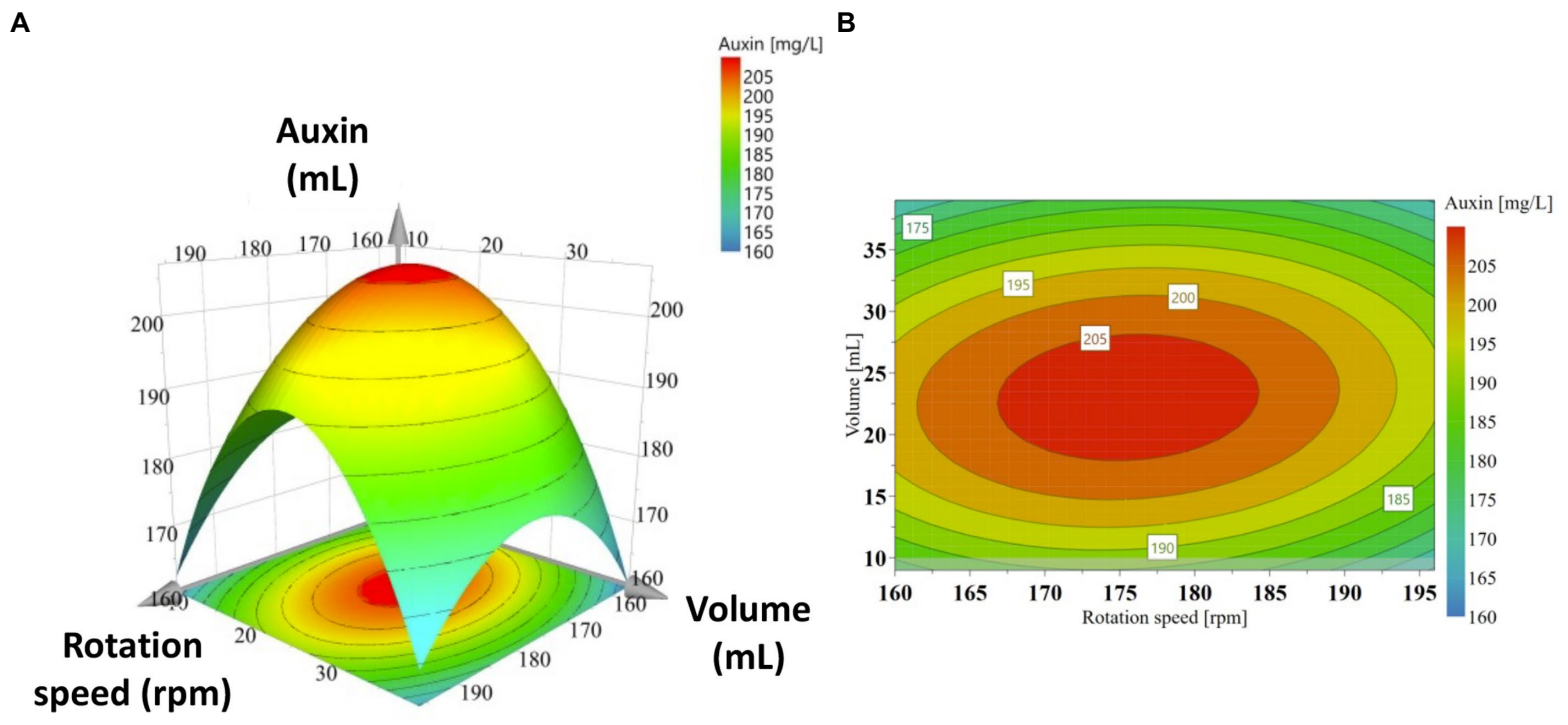
Response surface plots (3D, **A**) and contour plots (2D, **B**) of auxin production as a function of significant interactions between factors: rotation speed (*X*_1_) and medium volume (*X*_2_).

A response surface was also produced to determine each variable’s optimum level for maximum productivity response and the combined effect of rotation speed and medium-to-flask volume ratio on auxin production (Figure 4). The graphical representation was a useful method to visualize the relationship between the response and the experimental levels of each variable and the type of interaction between test variables to deduce the optimum conditions. Response surface and contour plots showed that the highest auxin production (>205 mg IAA_equ_/L) occurred when rotation speed ranges between 165 and 186 rpm and volume ranges from 17.25 to 29.25 mL. Above and below the ranges specified for rotation speed and volume, auxin production decreased, and lower values were obtained.

The objective of the application of RSM to C1 strain growth was to determine the levels of experimental factors which would allow obtaining the highest auxin production. The model allowed the prediction of an auxin production of 207.3 mg IAA_equ_/L when optimal growth conditions, that is, 176 rpm and 24 mL medium volume, were applied. To validate the predicted model, five experiments were thus conducted at these optimum growth conditions, and the results showed a close match (208.3 ± 0.4 mg IAA_equ_/L).

### Effect of cultivation conditions on auxin profile

**Figure 5:**
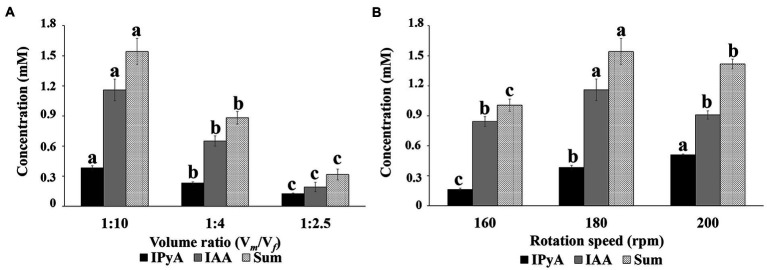
Results of the HPLC-MS analysis showing the effect of liquid-to-flask volume ratio **(A)** and rotation speed **(B)** on the conversion of tryptophan (Trp) to indole-3-acetic acid (IAA) *via* the indole-3-pyruvic acid (IPyA) pathway. All cultures were grown in YES + Trp medium at 25°C for 24 h. Data are means ± standard error and refer to cultures grown at 180 rpm **(A)** and a medium-to-flask volume ratio (V*_m_*/V*_f_*) of 1:10 **(B)**. The graphs show the molar concentration of the single compounds and the IAA + IPyA sum. Bars with no letter in common significantly differ at *p* ≤ 0.05 (Tukey HSD test).

Targeted metabolomics provided insights into the effect of the culture conditions on the levels of IAA and its main precursor, indole-3-pyruvic acid (IPyA; Figure 5). After 24 of growth at 180 rpm, IPyA accumulated in the culture medium at a concentration between 0.12 ± 0.0 mM (and V*_m_*/V*_f_* ratio of 1:2.5) and 0.5 ± 0.0 mM (V*_m_*/V*_f_* ratio of 1:10; Figure 5A).

At a V*_m_*/V*_f_* ratio of 1:10, we observed an increase in the IPyA concentration (from 0.2 ± 0.0 to 0.5 ± 0.0 mM; Figure 5B) and a decrease in the IAA/IPyA ratio (from 5.2 ± 0.1 to 1.8 ± 0.1; Table 5) by increasing the agitation speed from 160 to 200 rpm.

**Table 5:** Effect of medium-to flask volume ratio (V*_m_*/V*_f_*) and rotation speed (rpm) on the product selectivity (IAA/IPyA) and substrate consumption rate (final-to-initial Trp ratio).

Factor	Condition	Relative molar ratio	
		IAA/IPyA	Trp*_f_*/Trp*_i_*
*Volume ratio* (V*_m_*/V*_f_*)	1:10	3.0 ± 0.1^a^	1.1 ± 0.1*^b^*
	1:4	2.8 ± 0.1^b^	1.3 ± 0.1*^a^*
	1:2.5	1.5 ± 0.0^c^	1.2 ± 0.1*^ab^*
*Rotation speed* (rpm)	160	5.2 ± 0.1^A^	0.6 ± 0.0*^C^*
	180	3.0 ± 0.1^B^	1.1 ± 0.1*^A^*
	200	1.8 ± 0.1^C^	1.0 ± 0.1*^B^*

The superscript letters indicate similarities or significant differences between the values. Values with no letter in common significantly differ at *p* ≤ 0.05 (Tukey HSD test).

At constant agitation speed (180 rpm), we observed that the accumulation of IAA and IPyA were inversely correlated to the culture volume. The IAA/IPyA ratio increased (from 1.5 ± 0.0 to 3.0 ± 0.1) by decreasing the culture volume from 100 (V*_m_*/V*_f_* = 1:2.5) to 25 (V*_m_*/V*_f_* = 1:10; Table 5).

The sum of IAA and IPyA concentrations was affected by both parameters. It was maximal (1.5 ± 0.1) at a working volume of 25 mL in a 250 mL flask (V*_m_*/V*_f_* = 1:10) and an agitation speed of 180 rpm (Figure 5).

The change in tryptophan (Trp) concentration (the precursor of IPyA and IAA; Table 5) was not consistent with the total content of IAA + IPyA (Figure 5). In almost all culture conditions, the moles of products (IAA + IPyA) obtained per moles of the substrate (Trp) were not equal to 1. Notably, at V*_m_*/V*_f_* = 1:10 (25 mL) and 160 rpm, only part of the consumed Trp was converted to IAA and IPyA (Table 5). In contrast, increasing the agitation speed up to 180 or 200 rpm, the final concentration of Trp after 24 h of growth was higher or equal to that of the not inoculated broths (Table 5), indicating that higher agitation speeds (up to 180 rpm) promote the biosynthesis of both IAA (Table 4) and Trp (Table 5).

## Discussion

The production of phytohormones such as auxin is one of the central mechanisms beneficial bacteria use to enhance plant growth. Selection of the culture medium and optimization of the culture parameters that affect the biosynthesis of these compounds is thus pivotal to obtaining plant postbiotics based on auxin and plant growth-promoting molecules produced by PGPR. We previously demonstrated that selected *P. agglomerans* strains secrete a combination of metabolites that can act in synergy with auxins to induce adventitious root formation (Luziatelli et al., 2020c). Using *Pyrus communis* as a model system, we demonstrated that, in contrast to the synthetic hormone indole-3-butyric acid (IBA), the metabolites secreted by *P. agglomerans* C1 strain have an unusual auxin-like activity that could determine direct shoot regeneration without callus formation and earlier emergence of roots from stem tissues (Luziatelli et al., 2020c).

One of the major goals of this work was to analyze how to rationally develop plant biostimulants that fit within the definition of postbiotics (“*preparation of inanimate microorganisms and/or their components*”) and make the most of the biosynthetic capabilities of a selected PGPR strain integrating metabolomics and fermentation technologies. For this purpose, we first identified, by untargeted metabolomics, the culture medium that better stimulates the production of a more comprehensive array of metabolites with plant-promoting or biocontrol activity, including auxin/IAA. We then used a three-level-two-factor response surface methodology based on the central composite design (RSM-CCD) to optimize the auxin/IAA production on the selected medium. For this purpose, we set two parameters (rotation speed and medium liquid-to-flask volume ratio) that affect the aeration efficiency in shake-flask conditions as response factors. Moreover, to better understand the interaction between these two parameters, we used an iterative RSM-CCD approach to define the correct ranges of the factors. Finally, by targeted metabolomics, we analyzed the effect of the aeration efficiency of the culture medium on the IAA product selectivity and the metabolic flux of Trp to IAA through IPyA.

Results reported in Figure 1 indicated that, in the production of a plant postbiotic, the culture medium has a central role in the balance between the target compound (indole auxins) and other metabolites secreted by the microorganism. A PGPR can produce and release in the culture medium a wide array of compounds that can enhance or modulate the plant’s response to the exogenous auxins (Duca et al., 2014) or promote plant growth through different mechanisms (Olanrewaju et al., 2017). In this respect, the possibility of modifying the hypoxanthine/xanthine ratio shifting from LB to YES medium can be particularly interesting since these molecules interplay with the plant’s response to auxin, environmental stresses, and, in the case of hypoxanthine, purine savage (Ashihara et al., 2018).

Untargeted metabolomics also indicated that the shift from LB to YES had a positive effect on the accumulation of some *P. agglomerans* metabolites with antimicrobial activity that can indirectly affect the promotion of plant growth, preventing the development of plant pathogens (Glick, 1995). Among these compounds, we found: NS-5, a β-lactam antibiotic active against Gram-positive and Gram-negative bacteria (Papp-Wallace et al., 2011); methyl hexadecanoic acid, a fatty acid methyl ester (FAME) with antibacterial activity against Gram-negative (Thormar, 2010); indole-3-carbinol, a compound toxic to herbivores, insects and pathogens (Kim and Jander, 2007; Figure 2; Supplementary Table S1).

In summary, the metabolomics analysis indicated that the use of plant postbiotics, in alternative to IBA or other synthetic hormones, gives the advantage of obtaining a selected array of compounds with plant-promoting or biocontrol activity whose synthesis pattern in the natural environment is unpredictable and whose importance in plant-microbe interaction could not be easily recognized using conventional systems. At the same time, the use of microbial metabolites by PGP bacteria or fungi not included in the list of microbial biostimulants approved by the EU legislation 2019/1009 can offer an interesting opportunity for scientists and entrepreneurs to develop novel bio-based biostimulants with a well-defined composition and well-characterized activities.

Results reported in Figure 1 also indicated that the shift from the rich (LB) to the saline (YES) medium determined a reduction in the formation of indolic secondary metabolites, including indole-3-acetaldehyde and indole-3-carboxylic acid (Supplementary Table S2). The latter compound can play an important role in pathogen defense (Böttcher et al., 2014) but can interfere with auxin-signaling (Kumar et al., 2021). Comparative metabolomics indicated that, if the primary goal is the production of postbiotics with higher auxin/IAA levels, the YES medium can provide better results regarding IAA titer and product selectivity. With *P. agglomerans* strain C1, shifting from LB to YES medium, most of the Trp-derived indoles, 11 out of 14 compounds belonging to the indoles and derivatives cluster, decreased, and a 5.4-fold IAA titer increase occurred (Supplementary Table S2). These differences can be dependent on differences in the composition of the growth media in terms of carbon and nitrogen source (peptides in LB and sucrose and NH_4_^+^ ions in YES) and Mg^2+^ ion concentration (30–40 μM in LB and 1 mM in YES; Papp-Wallace and Maguire, 2008). Magnesium ions, in combination with thiamine pyrophosphate (ThDP), are an essential cofactor of IPDC, the key enzyme involved in IAA biosynthesis *via* the tryptophan-dependent IPyA pathway. Koga et al. (1992) demonstrated that, in the absence of ThDP and Mg^2+^, the active IPDC tetramers dissociate into inactive monomers and dimers. It can be postulated that the differences in the Mg^2+^ ion concentration can differentially modulate the IPCD activity and play a role in the differential accumulation of the Trp-derived indoles. The scientific literature provides extensive evidence that the magnesium availability in the fermentation media significantly affects the growth and metabolism of microbial cells (Walker, 1994). Our results showed that a culture medium with a higher Mg^2+^ content could be valuable for the microbial production of auxin/IAA through the IPyA pathway.

Many reports indicate that, in bacteria, auxin/IAA production is affected by several parameters, including medium components, growth temperature, incubation time, and medium pH (Apine and Jadhav, 2011; Balaji et al., 2012; Peng et al., 2014; Bhutani et al., 2018; Srisuk et al., 2018; Myo et al., 2019; Baliyan et al., 2021). Notably, none of these studies reported a detailed metabolomics analysis of the effect of these parameters on the production of other auxin-related compounds. On the other hand, no information is available on the effect of aeration efficiency on the microbial production of phytohormones such as auxin, whereas oxygen plays a crucial role in the growth and metabolism of aerobic and facultative anaerobic bacteria (Garcia-Ochoa et al., 2010; Seidel et al., 2021).

In this work, we demonstrated that the statistical optimization of the growth conditions was a powerful tool to optimize the auxin production by *P. agglomerans* strain C1 (Figure 4; Tables 2, 3). To the best of our knowledge, this is the first study investigating the effect of aeration efficiency on auxin production by plant growth-promoting bacteria. The statistical optimization of aeration efficiency, performed by varying the medium liquid-to-flask volume ratio (V*_m_*/V*_f_*) and rotation speed (rpm; Table 1), provided valuable insights into the effect of oxygen availability on IAA production by the *P. agglomerans* strain C1 (Tables 2, 3). From an agroecological perspective, this result also highlights the importance of oxygen availability on the plant-microbe’s interaction in the rhizosphere, phyllosphere, and endosphere.

Our results indicated that the parameters that directly affect aeration efficiency in shake flask conditions (rotation speed and liquid-to-flask volume ratio) play an essential role in auxin/IAA production, which was improved from 3.1 mg IAA_equ_/L (CCD1, rotation speed 140 rpm, 1:2.5 V*_m_*/V*_f_*) to 208.1 mg IAA_equ_/L (CCD2, rotation speed 180 rpm, 1:10 V*_m_*/V*_f_*; Table 2; Figure 3). In agreement with this observation, higher IAA values were achieved using a 1:10 liquid-to-flask volume ratio at all agitation regimes (Table 2; Figure 3B). A higher amount of auxin/IAA was obtained when the oxygen availability was higher because of higher rotation speed and lower medium liquid-to-flask volume ratio (Tables 2, 3). However, data showed low tolerance of strain C1 to mechanical stress: IAA concentration decreased when the rotation speed was higher than 180 rpm (Table 3).

The polynomial equation indicated that the two independent variables, rotation speed (*X*_1_ and *X*_1_^2^) and medium volume (*X*_2_ and *X*_2_^2^), had a negative effect on the response (Table 4). In contrast, the interactive effect of these variables (*X*_1_*X*_2_) positively affected the auxin production (Table 4). The equation also indicated that the coefficient of the quadratic *X*_2_ was larger than that of *X*_1_^2^, which means that the medium volume, and therefore the medium volume-to-flask ratio, had a greater effect on the auxin production than the rotation speed. This information was corroborated by the curvature of the 3D response surface that was more pronounced on the “Medium Volume” axis (Figure 4A). Similar evidence was also obtained by the 2D contour plot in which the contours around the stationary point were elliptical and elongated on the rotation speed axis (Figure 4B). Both plots indicated that: (1) at the extreme values of both variables, auxin/IAA concentration was low; (2) a slight change of the response value required a more significant move along the rotation speed axis than on the medium volume axis.

Validation carried out to verify the accuracy of the model showed that the predicted value (207.3 mg IAA_equ_/L) agreed well with the experimental value (208.3 ± 0.4 mg IAA_equ_/L). The application of the RSM through CCD allowed us to obtain the maximum auxin concentration at a medium liquid-to-flask volume ratio of 1:10 and a rotation speed of 176 rpm. The auxin concentration obtained under optimized conditions (208.3 ± 0.4 mg IAA_equ_/L) was about 40% higher compared to the growth conditions previously described (Luziatelli et al., 2020b).

Targeted metabolomics highlighted some features of the *P. agglomerans’* IAA biosynthetic metabolism, which were not addressed by the RSM analysis underlining the importance of integrating the two methodologies. Data in Table 5 indicated that the culture conditions affected the flux of Trp to IAA through IPyA. At a rotary speed of 180 rpm, the product selectivity (IAA/IPyA molar ratio) had a 2-fold increase reducing the medium-to-flask volume ratio from 1:2.5 to 1:10 (higher aeration efficiency; Table 5). Surprisingly, at the lower medium-to-flask volume ratio (1:10), the product selectivity decreased about 2.9-fold when the rotation speed was increased from 160 to 200 rpm (Table 5). Thus, independently from the final concentration of IAA, it was evident that the mechanical and oxidative stresses associated with the increase in the rotary speed affected the conversion of Trp to IAA.

Results of targeted metabolomics indicated that an increase in the rotation speed determined an accumulation of IPyA in the culture medium (Figure 5B) and a decrease in the IAA product selectivity (Table 5, column IAA/IPyA). The increased difference between IAA and IAA + IPyA (Figure 5B) also indicated that the moles of IPyA converted to IAA decreased with the increase in the rotation speed from 180 to 200 rpm. Since no significant difference in the IAA level was observed between rotation speeds 160 and 200 rpm, the variation in the total amount of IAA + IPyA was strictly related to a difference in the accumulation of IPyA (Figure 5B). The higher flux of Trp in the IAA biosynthetic pathway and the lower conversion rate of IPyA to IAA occurring when the rotation speed was increased from 160 to 180 rpm could be due to a difference in the availability of the cofactors of the key enzyme of the IPyA pathway: pyridoxal-5-phosphate (PLP) for L-tryptophan/aromatic amino acid aminotransferase (AAT); thiamin diphosphate for indole-3-pyruvate decarboxylase (IPDC); NAD(H)^+^ for indole-3-acetaldehyde (IAAld) dehydrogenase/oxidase (AAO). Based on the literature (Lakaye et al., 2004; Taboada et al., 2008; Tramonti et al., 2021; Yuan et al., 2021), we expected, also in *P. agglomerans*, that the levels of these cofactors could be higher when the dissolved oxygen is not a limiting factor and its replenishment satisfies metabolic oxygen demands. However, the availability of these cofactors for the enzymes involved in the IAA synthesis depends upon the number of competitive reactions that consume the same cofactors and are active under the same growth conditions. In future research, we intend to combine metabolomics and RSM analysis to explore the correlation between IAA product selectivity and cofactors availability and improve the fermentation strategies focusing on both IAA yield and product selectivity.

Whereas in all *P. agglomerans* strains described so far (including strain C1), the production of high levels of IAA is dependent upon the addition of Trp to the culture medium, our data indicated that, under our experimental conditions, strain C1 synthetized Trp and released part of it in the culture medium (Table 5, column Trp*_f_*/Trp*_i_*). Interestingly, the highest IAA product selectivity was obtained when the residual Trp level was lower (Table 5, rotation speed of 160 rpm). Considering that the first step of the major pathways of Trp degradation involves PLP-dependent enzymes (Cellini et al., 2020), it can be postulated that the flux of Trp versus IPyA is modulated by the affinity of the AAT versus the substrate (Trp) and the cofactor (PLP) and the intracellular concentration of both. The highest IAA product selectivity obtained when the Trp biosynthesis is limited (Table 5, rotation speed of 160 rpm) can indicate that redirecting the Trp metabolic flux is pivotal, either in laboratory conditions or in natural environments, to modulate the IAA production by PGPRs.

The levels of auxin/IAA obtained in this study are lower than those reported for *P. agglomerans* PVM (Apine and Jadhav, 2011), *Klebsiella* SN 1.1 (Chaiharn and Lumyong, 2011), *Enterobacter* sp. DMKU-rp206 strain (Srisuk et al., 2018) and *Enterobacter* sp. P-36 (Luziatelli et al., 2021), but are indeed higher than those obtained upon optimization of IAA production by *Bacillus* strains isolated from *Vigna radiata* (Bhutani et al., 2018), *Streptomyces* sp. VSMGT1014 (Harikrishnan et al., 2014), *Burkholderia seminalis* S52 (Tallapragada et al., 2015), or *Pseudomonas putida* sp. Rs-198 strain (Peng et al., 2014). For *P. agglomerans* strain PVM (Apine and Jadhav, 2011), the higher levels of IAA were produced on a medium containing animal-derived ingredients (meat), which are not compliant with EU rules on fertilizing products.

## Conclusion

Integration of RSM with metabolomics, which provides comprehensive information on the cellular response to external stimuli, can be valuable for optimizing various microbial products. In this study, we provided evidence that, in developing auxin-enriched plant postbiotics, the combined use of RSM and untargeted and targeted metabolomics can help discover new properties of PGPBs and take advantage of their metabolic capabilities. Postbiotics allow the rational design of cocktails of metabolites associated with PGP traits. The separate production of metabolites can be a valuable strategy in synthesizing molecules whose biosynthetic pathways compete in the cell for ATP, cofactors (auxins and gibberellins), or precursors (auxins and phenylpropanoids). Metabolites synthesized under different growing conditions or from different PGPBs can be produced separately and appropriately mixed to obtain tailor-made products for different crops and growing conditions. This study provides guidance for the sustainable development and optimization of postbiotics for agriculture.

## Data availability statement

The original contributions presented in the study are included in the article/Supplementary material, further inquiries can be directed to the corresponding authors.

## Author contributions

FM, FL, and MR contributed to the conception and design of the study, wrote the first draft of the manuscript, and wrote and edited the final version of the manuscript. FM, FL, PB, AF, VM, and MR contributed to defining the methodology and investigation. FM, FL, PB, and MR contributed to software analysis and data validation. All authors contributed to the article and approved the submitted version.

## Funding

This study was carried out within the Agritech National Research Center and received funding from the European Union Next-GenerationEU [Piano Nazionale di Ripresa e Resilienza (PNRR)—Missione 4 Componente 2, Investimento 1.4—D.D. 1032 17/06/2022 and CN00000022].

## Conflict of interest

The authors declare that the research was conducted in the absence of any commercial or financial relationships that could be construed as a potential conflict of interest.

## Publisher’s note

All claims expressed in this article are solely those of the authors and do not necessarily represent those of their affiliated organizations, or those of the publisher, the editors and the reviewers. Any product that may be evaluated in this article, or claim that may be made by its manufacturer, is not guaranteed or endorsed by the publisher.

## Author disclaimer

This manuscript reflects only the authors’ views and opinions, neither the European Union nor the European Commission can be considered responsible for them.
