# Supplementary material for: Optimization of the growth conditions through response surface methodology and metabolomics for maximizing the auxin production by Pantoea agglomerans C1
Source: Front Microbiol. 2023 Mar 8;14:1022248. doi: 10.3389/fmicb.2023.1022248 (PMC10030972; doi:10.3389/fmicb.2023.1022248)
Supplement: Supplementary file 1 [file Data_Sheet_1.PDF]

Rational bioprocess design for production of a plant-promoting postbiotic

Francesca Melini [1,2], Francesca Luziatelli [1]*, Paolo Bonini [3], Anna Grazia Ficca [1], Valentina Melini [2] and Maurizio Ruzzi [1]*

[1]Department for Innovation in Biological. Agrofood and Forest Systems. University of Tuscia. Viterbo, Italy
[2]CREA Research Centre for Food and Nutrition. Via Ardeatina 546. I-00178 Rome, Italy
[3]OloBion–OMICS LIFE LAB. Barcelona, Spain

*** Correspondence:**
Francesca Luziatelli
f.luziatelli@unitus.it

Maurizio Ruzzi
ruzzi@unitus.it

Supplementary Material

TABLE S1 | Results of the ChemRICH enrichment analysis for the altered metabolites secreted *by P. agglomerans* C1 shifting from LB to YES medium. Data refer to the relative abundance of metabolites produced in both culture media.

Cluster name	Cluster size	*p*-values	FDR	Altered metabolites	Increased	Decreased
Carboxylic acids and derivatives	42	2.2E-16	3.3E-15	28	7	16
Indoles and derivatives	15	5.6E-16	4.2E-15	14	3	11
Organooxygen compounds	20	1.3E-09	6.4E-09	15	7	7
Imidazopyrimidines	6	0.00000002	7.4E-08	6	2	3
Fatty Acyls	11	0.0000045	0.000013	8	4	4
Benzene and substituted derivatives	13	0.0000078	0.000019	6	2	3
Steroids and steroid derivatives	5	0.00034	0.00062	4	2	2
Quinolines and derivatives	5	0.00035	0.00062	4	3	1
Diazanaphthalenes	4	0.00037	0.00062	3	1	2
Phenols	4	0.0011	0.0017	3	0	3
Prenol lipids	8	0.0023	0.0032	4	1	3
Diazines	5	0.013	0.016	3	2	1
Organonitrogen compounds	3	0.014	0.016	1	0	1
Glycerophospholipids	3	0.096	0.1	2	0	0
Azoles	3	0.16	0.16	1	1	0

TABLE S2 | Relative abundance of strain C1 metabolites belonging to the indoles and derivatives cluster. Compounds, whose relative abundance significantly increased (≥2-fold) shifting cells from LB to YES medium, are highlighted in green.

Compound name	p-value	effect_size	Cluster	e-direction	efs	FDR
**3-Indoleacetic acid**	**0.000007**	**5.40**	**Indoles and derivatives**	**up**	**5.4**	**0.0014**
**Indole-3-carbinol**	**0.00000043**	**3.95**	**Indoles and derivatives**	**up**	**3.9**	**0.000086**
Indoline	0.029	1.17	Indoles and derivatives	no change	1.1	1
Indalpine	0.15	1.10	Indoles and derivatives	no change	1	1
Frovatriptan	0.0062	0.86	Indoles and derivatives	no change	1.2	0.81
1H-Indole-3-carboxaldehyde	0.00000015	0.64	Indoles and derivatives	no change	1.6	0.00003
Indole	0.0017	0.59	Indoles and derivatives	no change	1.7	0.26
Indole-3-acrylic acid	0.0013	0.28	Indoles and derivatives	down	3.6	0.21
Sempervilam	0.0024	0.27	Indoles and derivatives	down	3.7	0.36
SU5402	0.024	0.27	Indoles and derivatives	down	3.7	1
Caulersin	0.014	0.18	Indoles and derivatives	down	5.6	1
Indoleacetaldehyde	0.000082	0.12	Indoles and derivatives	down	8.5	0.015
Secofascaplysin A	0.021	0.06	Indoles and derivatives	down	16	1
Violacein	0.0036	0.05	Indoles and derivatives	down	19	0.5
Indole-3-carboxylic acid	0.00097	0.04	Indoles and derivatives	down	24	0.16
